# Lipid metabolism-related gene prognostic index (LMRGPI) reveals distinct prognosis and treatment patterns for patients with early-stage pulmonary adenocarcinoma

**DOI:** 10.7150/ijms.71267

**Published:** 2022-03-28

**Authors:** Aimin Jiang, Xue Chen, Haoran Zheng, Na Liu, Qianqian Ding, Yimeng Li, Chaoxin Fan, Xiao Fu, Xuan Liang, Tao Tian, Zhiping Ruan, Yu Yao

**Affiliations:** Department of Medical Oncology, The First Affiliated Hospital of Xi'an Jiaotong University, Xi'an, Shaanxi, People's Republic of China.

**Keywords:** Lipid metabolism, Prognosis, Prognostic model, Immunotherapy, Lung adenocarcinoma

## Abstract

**Background:** Lipid metabolism plays a pivotal role in cancer progression and metastasis. This study aimed to investigate the prognostic value of lipid metabolism-related genes (LMRGs) in early-stage lung adenocarcinoma (LUAD) and develop a lipid metabolism-related gene prognostic index (LMRGPI) to predict their overall survival (OS) and treatment response.

**Methods:** A total of 774 early-stage LUAD patients were identified from The Cancer Genome Atlas (TCGA, 403 patients) database and Gene Expression Omnibus (GEO, 371 patients) database. The non-negative Matrix Factorization (NMF) algorithm was used to identify different population subtypes based on LMRGs. The Least Absolute Shrinkage and Selection Operator (LASSO) and multivariate Cox regression analyses were used to develop the LMRGPI, with receiver operating characteristic (ROC) curves and concordance index being used to evaluate its performance. The characteristics of mutation landscape, enriched pathways, tumor microenvironment (TME), and treatment response between different LMRGPI groups were also investigated.

**Results:** We identified two population subtypes based on LMRGs in the TCGA-LUAD cohort, with distinct prognosis, TME, and immune status being observed. LMRGPI was developed based on the expression levels of six LMRGs, including *ANGPTL4*, *NPAS2*, *SLCO1B3*, *ACOXL*, *ALOX15*, and *B3GALNT1*. Higher LMRGPI was correlated with poor OS both in TCGA and GSE68465 cohorts. Two nomograms were established to predict the survival probability of early-stage LUAD, with higher consistencies being observed between the predicted and actual OS. Higher LMRGPI was significantly correlated with more frequent TP53 mutation, higher tumor mutation burden (TMB), and up-regulation of CD274. Besides, patients with higher LMRGPI presented unremarkable responses for gefitinib, erlotinib, cisplatin, and vinorelbine, while they tend to have a favorable response for immune checkpoint inhibitors (ICIs). The opposite results were observed in the low-LMRGPI group.

**Conclusions:** We comprehensively investigated the prognostic value of LMRGs in early-stage LUAD. Given its good prognostic ability, LMRGPI could serve as a promising biomarker to predict the OS and treatment response of these patients.

## Introduction

Lung cancer is one of the most common incident cancers and the leading cause of cancer-related death worldwide 1. As the most predominant pathological subtype, lung adenocarcinoma (LUAD) makes up more than 40% of lung cancer cases 2, 3. In recent decades, although we have made promising progress in the screening, diagnosis, and management of LUAD patients, it remains a lethal disease because a large fraction of patients is diagnosed at the advanced disease stage 4, 5. Approximately 30% of LUAD patients are diagnosed at early stage with limited disease symptoms, with surgical resection and post-operation adjuvant therapy being recommended for these patients according to various protocols 6, 7. Adjuvant treatment is not required for stage IA patients with negative tumor margins 6, 8. However, stage IB patients with high-risk factors and stage IIA patients need to receive adjuvant chemotherapy to avoid disease relapse as recommended by National Comprehensive Cancer Network (NCCN) Guidelines 8. Although surgery and adjuvant treatment could bring remarkable survival benefits for these individuals, some patients still cannot escape the fate of disease recurrence within five years 6, 9. Therefore, effective biomarkers that could monitor disease recurrence and progression are urgently needed for patients with early-stage LUAD.

Lipids, a large class of metabolites composed of different fatty acids 10, are essential components of the biological membranes and structural units that make up cells 11. Besides, they are also used for energy storage and metabolism and serve as crucial signaling molecular roles in most cellular activities 11. Altered cellular metabolism and energetics are recognized hallmarks of cancer cells. Accumulating evidence elucidated that lipid metabolism disorder in the tumor microenvironment (TME) is significantly correlated with the malignant phenotypes of cancer cells 11. For instance, Hall et al. reported that MYC drives the production of specific eicosanoids, which are critical for lung cancer cell survival and proliferation 12. This phenomenon indicated that MYC expression drives aberrant lipid metabolism in lung cancer. Furthermore, Zhang et al. found that knockdown of MGLL (a key enzyme in lipid metabolism) inhibits the proliferation and metastasis of LUAD cell lines, supporting that lipid metabolism plays a pivotal role in LUAD progression and metastasis 13. As we all know, TME also serves an important role in cancer progression, metastasis, immune evasion, and treatment resistance.

The crosstalk between altered lipid metabolism and TME can strongly impact other cancer hallmarks 14. Different components of TME have distinct metabolism programs 14. Lipid metabolism reprogramming of different immune cells could change the biological behavior of the tumor and affect the antitumor immune response 14, 15. Therefore, targeting lipid metabolism is considered as a new strategy for malignancy treatment. This study systematically evaluated the prognostic value of lipid metabolism-related genes (LMRGs) in early-stage LUAD. In this study, we developed a lipid metabolism-related gene prognostic index (LMRGPI) that based on the expression levels of six LMRGs to predict the prognosis and treatment response for early-stage LUAD patients. An independent external validation cohort was also used to evaluate its predictive ability and risk stratification ability. Besides, we also identified two different LMRGs subtypes that have very different prognosis and immune characteristics via non-negative Matrix Factorization (NMF) algorithm. We believe our findings will provide potential biomarkers and therapeutic targets for these individuals.

## Materials and methods

### Raw data acquisition and processing

The transcriptional, mutation and clinical data of 403 stage I-II LUAD samples and 43 adjacent tissues were downloaded from the TCGA database. Because there were no progression-free survival (PFS) and disease-free survival (DFS) records in the TCGA database, we obtained these data from UCSC Xena (https://xena.ucsc.edu/). There were three formats (count, FPKM, and FPKM-UQ) of RNA-seq data, and we selected the second one for further analysis. The human.gtf file was adopted to raw matrix annotation. Furthermore, the GSE68465 cohort was also downloaded from the Gene Expression Omnibus (GEO) (https://www.ncbi.nlm.nih.gov/) database, an independent external validation cohort, which including transcriptional data and clinical information of 371 early-stage LUAD patients. As previously reported, 776 LMRGs were obtained from the Molecular Signature Database v. 7.0 (MSigDB, http://www.gsea-msigdb.org/gsea/msigdb/). We used the “VLOOK UP” function in Microsoft Excel to match the gene expression matrix with clinical data according to their unique ID number to generate the merged matrix for later analysis. The detailed clinicopathological characteristics of patients in the TCGA and GSE68465 cohorts is presented in **Table 1**.

### DELMRGs identification and functional enrichment analysis

First, we adopted intersecting analysis between the gene expression matrix of early-stage LUAD patients from the TCGA-LUAD cohort and the extracted LMRGs. Then, differential expression analysis was performed to filter differentially expressed lipid metabolism-related genes (DELMRGs) between early-stage LUAD and normal samples by using the R software, “limma” package, with |log_2_(Foldchange)| >1.0 and false discovery rate (FDR)< 0.05 being used as cut-off value. The volcano plot and heatmap were also generated to visualize the distribution of the identified DELMRGs using R software, “ggplot2” and “pheatmap” packages. Subsequently, Kyoto Encyclopedia of Genes and Genomes (KEGG) and Gene Ontology (GO) analyses were exploited to investigate the most significantly enriched pathways and biological processes of the DELMRGs using R software, “clusterProfiler” package.

### Sample clustering using NMF algorithm

After differential expression analysis, intersected analysis was performed to identify common DELMRGs in the TCGA-LUAD and GSE68465 cohorts. Then, NMF was carried to divide patients into different subtypes according to the following steps: (a) the univariate Cox regression analysis was performed to identify potential prognostic DELMRGs via R software, “survival” package; (b) sample clustering through “brunet” method in R software, “NMF” package; (c) according to parameters such as cophenetic, dispersion, silhouette, and sparseness, the optimal number of the cluster was identified to classify patients into different subtypes; and (d) the consensus heatmap was generated in accordance with the above optimal cluster number to view the distribution characteristic among different subtypes.

Then, we also explored the relationship between different clusters and the prognosis of patients with early-stage LUAD, including OS, PFS, and DFS. The Kaplan-Meier survival curves were generated to depict the survival difference via R software, “survival” and “survminer” packages. The log-rank test was used to evaluate the statistical difference. Besides, the MCPcounter algorithm 16 was adopted to estimate the infiltration of the immune cells between different clusters. According to the previous study 17, six immune subtypes play tumor-promoting or suppressive effects in human cancer. We also investigated the association between different clusters and immune subtypes.

### LMRGPI construction and validation

We used the TCGA-LUAD cohort to develop the LMRGPI to stratify patients into different risk groups and predict their OS and treatment response. The GSE68465 cohort was set as an independent external validation cohort to assess the performance of LMRGPI. First, we performed univariate Cox regression analysis to identify potential prognostic LMRGPI for early-stage LUAD. Variables with a P value<0.05 were selected into the Least Absolute Shrinkage and Selection Operator (LASSO) regression analysis to reduce the number of genes in the final risk model through R software, “glmnet” package. Ultimately, genes in the LASSO regression were selected into the multivariate Cox regression analysis and therefore constructed the LMRGPI according to the following formula:



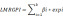



In the formula, “βi” represents the coefficient of the selected LMRG in the multivariate Cox analysis and “expi” refers to its expression value. Subsequently, all patients were divided into high- and low-LMRGPI groups according to the median value of LMRGPI. Survival curve and risk plot were generated to visualize the survival difference and status for each patient. Besides, the receiver operating characteristic (ROC) curve was also adopted to evaluate the performance of LMRGPI in predicting 1-, 3-, and 5-year OS of early-stage LUAD patients via R software, “survival”, “survminer”, and “timeROC” packages. Likewise, we performed the above analyses in the GSE68465 cohort to confirm whether the LMRGPI could be a potential prognostic factor by dividing patients into two groups according to the median of the LMRGPI in the TCGA cohort. Furthermore, cBioPortal (http://www.cbioportal.org/) database was exploited to summarize the mutation landscape of the identified LMRGs in the multivariate Cox analysis, with the “OncoPrint” module being used for visualization. Meanwhile, the Human Protein Atlas (HPA, https://www.proteinatlas.org/) was also adopted to analyze their protein expression level, and the original immunohistochemistry (IHC) Figures were obtained for further analysis. Next, we performed the univariate and multivariate Cox analyses in TCGA and GSE68465 cohorts to determine whether LMRGPI could be an independent prognostic factor for early-stage LUAD compared with other common clinicopathological parameters, such as age, gender, disease stage, T stage, and N stage.

### Nomogram development and evaluation

Then, we used R software, “rms” and “regplot” packages to develop two nomograms to illustrate each patient's 1-, 3-, and 5-year survival probability by integrating LMRGPI and common clinicopathological variables. Calibration curves were also generated to evaluate the consistency between the predicted and the actual OS. Furthermore, the Kaplan-Meier survival curve and ROC curve were also used to compare the discrimination ability of LMRGPI and other preexisting prognostic scores in predicting the OS of early-stage LUAD. Additionally, we also calculated their concordance index (C-index) and RMS values through R software, “rms” and “survcomp” packages to further assess their predictive ability.

### Clinical relevance, mutation landscape, and enrichment analysis between high- and low-LMRGPI groups

Next, we investigated the relationship between LMRGPI and clinicopathological characteristics, the identified clusters by NMF, and the previously defined immune subtypes using R software, “ComplexHeatmap” package. Two waterfall plots were generated to explore the detailed gene mutation characteristics between high- and low-LMRGPI groups via R software, “maftools” package. Gene set enrichment analysis (GSEA) was then performed to identify the most significantly enriched pathways between high- and low-LMRGPI groups using R software, “clusterProfiler” package. We used “c2.cp.kegg.v7.4.symbols.gmt” as a reference gene set and visualized the top five pathways in different groups.

### Immune cells infiltration and immune function status between high- and low-LMRGPI groups

Then, single-sample gene set enrichment analysis (ssGSEA) 18 was adopted to estimate the infiltrating score of immune cells and the activity of immune-related pathways using R software, “GSVA” and “GSEABase” packages. The Wilcoxon rank-sum test was used to compare the statistical difference between high- and low-LMRGPI groups. Besides, we also investigated the correlation between LMRGPI and immune cells infiltration, tumor mutation burden (TMB), and immune checkpoint inhibitors (ICIs) related genes expression levels.

### Chemotherapeutic and immunotherapy response rates between high- and low-LMRGPI groups

We then calculated the half inhibitory concentration (IC_50_) of commonly used antitumor drugs in the TCGA-LUAD dataset via R software, 'pRRophetic' package 19 to evaluate the clinical utility of LMRGPI for the treatment of early-stage LUAD. Meanwhile, the Wilcoxon signed-rank test was utilized to compare the difference in the IC_50_ between low- and high-LMRGPI groups. To further investigate the prognostic value of LMRGPI in predicting the OS of patients treated with ICIs, we downloaded the gene expression matrix and survival data of the IMvigor 210 cohort 20 and performed survival analysis. Besides, we calculated the area under the curve (AUC) of LMRGPI in predicting 1-, 3-, and 5-year OS of patients in the IMvigor 210 cohort. Tumor Immune Dysfunction and Exclusion (TIDE, http://tide.dfci.harvard.edu/) algorithm can predict anti-PD1 and anti-CTLA4 response across several melanoma datasets and a limited dataset of non-small cell lung cancer (NSCLC) 21. The TIDE score could help oncologists choose patients who are more suitable for ICIs therapy. In prospective clinical trials, the TIDE score will be of great significance in immunotherapy decision-making 21. With the help of the TIDE online webserver, we predicted the response rate of immunotherapy in high- and low-LMRGPI groups. Furthermore, we also explored the correlation between LMRGPI and TIDE score, microsatellite instability (MSI), immune exclusion score, and immune dysfunction score.

### Statistical analysis

The statistical difference between the categorical variables was detected by the Chi-square test. The non-parameter Wilcoxon rank-sum test was used to examine the relationship of continuous variables between the two groups. The LASSO regression and Cox regression analyses were used for LMRGPI development. Kaplan-Meier survival analysis was used to test the survival difference between different risk groups. A log-rank test was adopted to examine the statistical difference. A two-sided P-value < 0.05 was considered significant. All analyses were conducted in R software (version 3.6.3) for windows 64.0.

## Results

### DELMRGs identification and functional enrichment analysis

**Figure 1:**
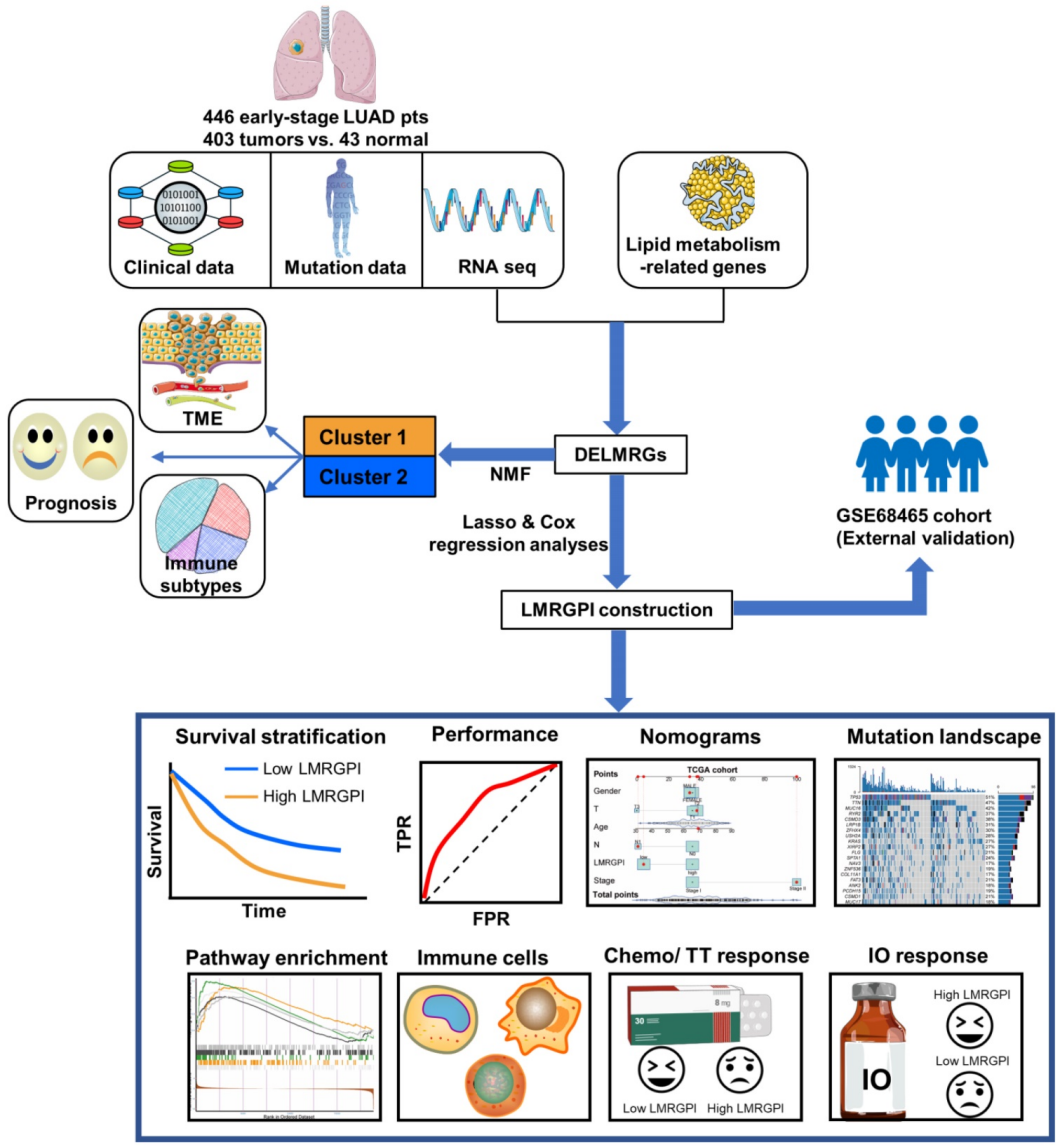
** Flow chart of the study.** LUAD, lung adenocarcinoma; TME, tumor microenvironment; NMF, non-negative Matrix Factorization; DELMRGs, differentially expressed lipid metabolism-related genes; LMRGPI, lipid metabolism-related gene index; TT, targeted therapy; IO, immunotherapy.

**Figure 2:**
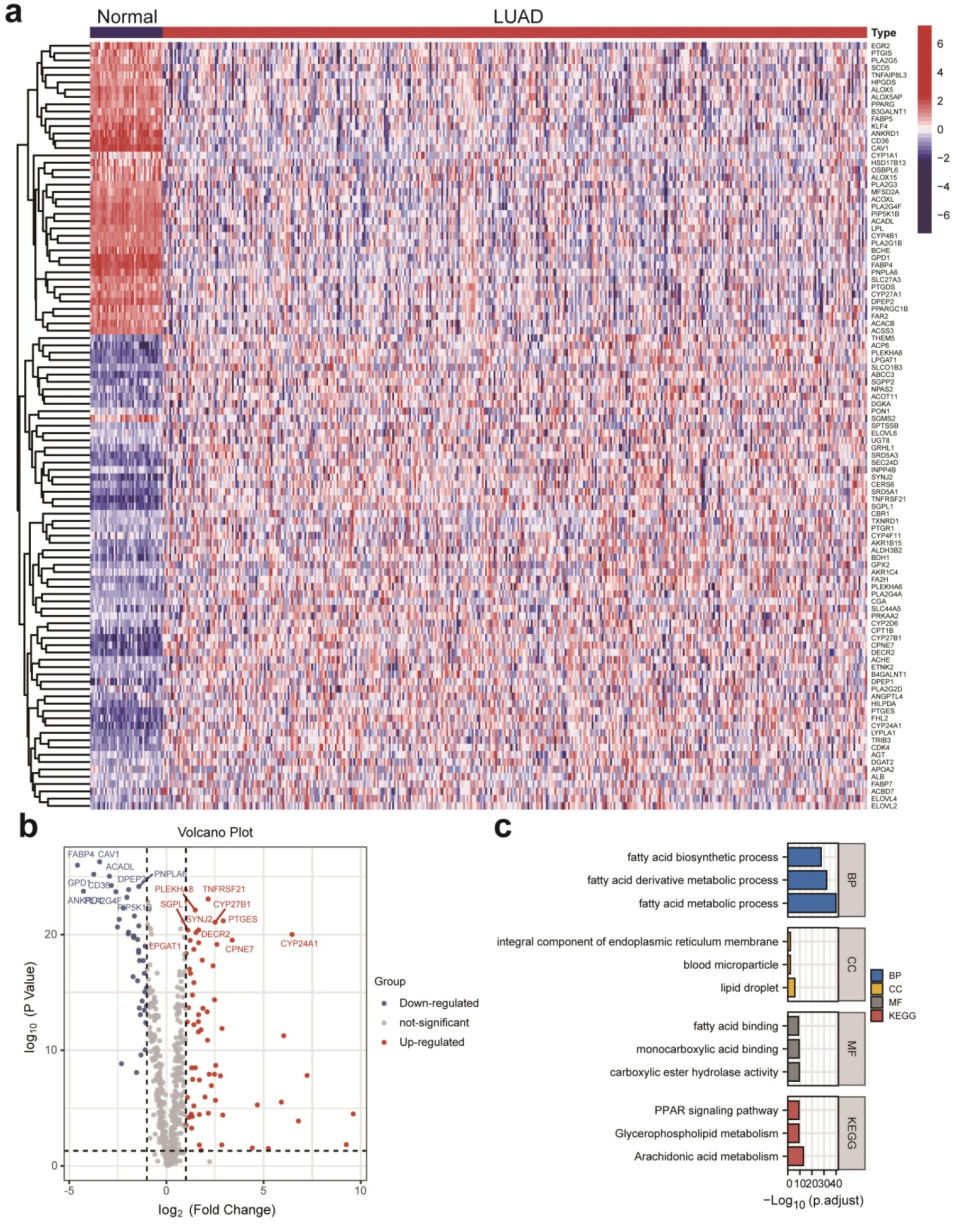
** DELMRGs identification of early-stage LUAD. (a)** The heatmap to show the DELMRGs between LUAD and normal samples. A total of 105 DELMRGs were identified through differential expression analysis (64 up-regulated and 51 down-regulated genes). **(b)** The volcano plot to show the up-regulated and down-regulated DELMRGs. **(c)** GO and KEGG enrichment analysis of the identified DELMRGs. DELMRGs, differentially expressed lipid metabolism-related genes; LUAD, lung adenocarcinoma; GO, Gene Ontology; KEGG, Kyoto Encyclopedia of Genes and Genomes; BP, biological process; CC, cellular component; MF, molecular function.

The detailed study process of this study is illustrated in **Figure 1**. There were 752 LMRGs in the TCGA-LUAD cohort after matching the gene expression matrix and LMRGs list (776 genes). A total of 105 genes were identified as DELMRGs after differential expression analysis (**Figure 2a**). Of these, 64 were up-regulated genes, while 51 were down-regulated genes (**Figure 2b**). Next, we conducted GO and KEGG enrichment analyses to investigate the most significantly enriched biological processes and pathways of the identified DELMRGs. Not surprisingly, GO analysis revealed that the DELMRGs were mainly enriched in the biological process that involved fatty acid metabolism (**Figure 2c**). KEGG analysis indicated that the DELMRGs were mainly enriched in the PPAR signaling pathway, glycerophospholipid metabolism pathway, and arachidonic acid metabolism pathway (**Figure 2c**).

### Different molecular subtypes identification based on DELMRGs

**Figure 3:**
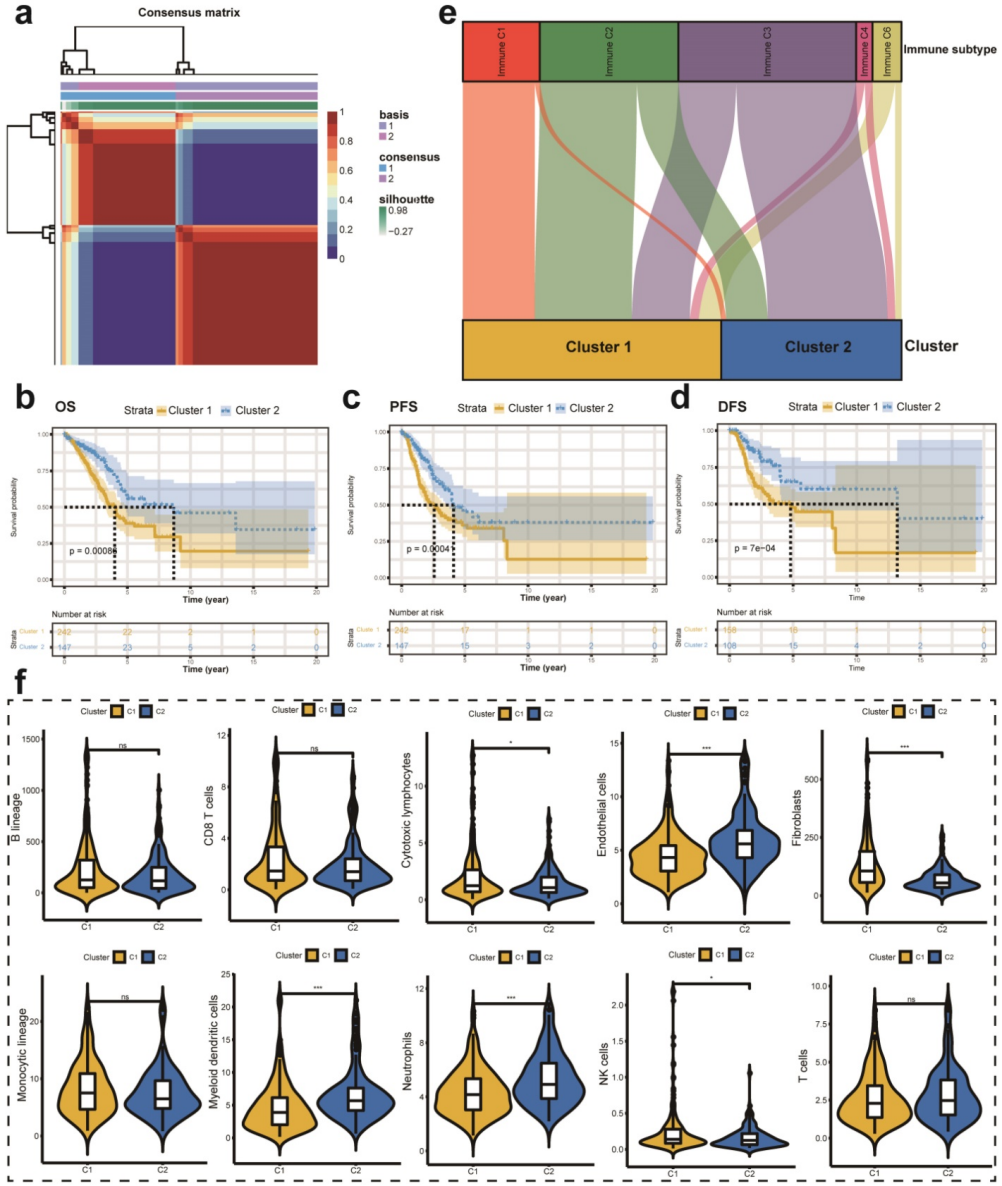
** Different LMRGs subtype identification and clinical relevance analysis. (a)** Two different subtypes were identified via the NMF algorithm. **(b-d)** The relationship between different subtypes and OS **(b)**, PFS **(c)**, and DFS **(d)** of early-stage LUAD. **(e)** Sankey plot to show the association between different subtypes and immune subtypes. **(f)** TME composition between different subtypes. LMRGs, lipid metabolism-related genes; NMF, non-negative Matrix Factorization; OS, overall survival; PFS, progression-free survival; DFS, disease-free survival; LUAD, lung adenocarcinoma; TME, tumor microenvironment; ns represents no statistical significance; * represents *P*<0.05; *** represents *P*<0.001.

First, we performed univariate Cox analysis to identify the most significant prognostic LMRGs in the TCGA-LUAD cohort. Then, we conducted NMF to divide patients into different clusters according to relevant parameters. In this analysis, we observed that the optimal number of clusters is two according to cophenetic, dispersion, silhouette, sparseness, and so on (**Figure S1a**). **Figure 3a** showed the expression level of LMRGs related to the prognosis of patients with early-stage LUAD in different clusters. Besides, we compared the OS, PFS, and DFS between different clusters. Better OS, PFS, and DFS were identified with patients in cluster 2 than in cluster 1 (**Figure 3b-d**). Besides, we also investigated the relationship between different immune subtypes and clusters via the Sankey plot. It showed that patients in cluster 1 are mainly classified into Immune C1 (wound healing), Immune C2 (IFN-gamma dominant), and Immune C6 (TGF-beta dominant) subtypes (**Figure 3e**). On the contrary, patients in cluster 2 are mainly classified into Immune C3 (inflammatory) subtype (**Figure 3e**). The MCPcounter algorithm was used to estimate the infiltration of the immune cells in different clusters. We found that the infiltration levels of cytotoxic lymphocytes, fibroblasts, and NK cells were significantly higher in cluster 1 than in cluster 2 (**Figure 3f**). However, cluster 2 had a higher infiltration level of endothelial cells, myeloid dendritic cells, and neutrophils (**Figure 3f**).

### LMRGPI construction and validation

**Figure 4:**
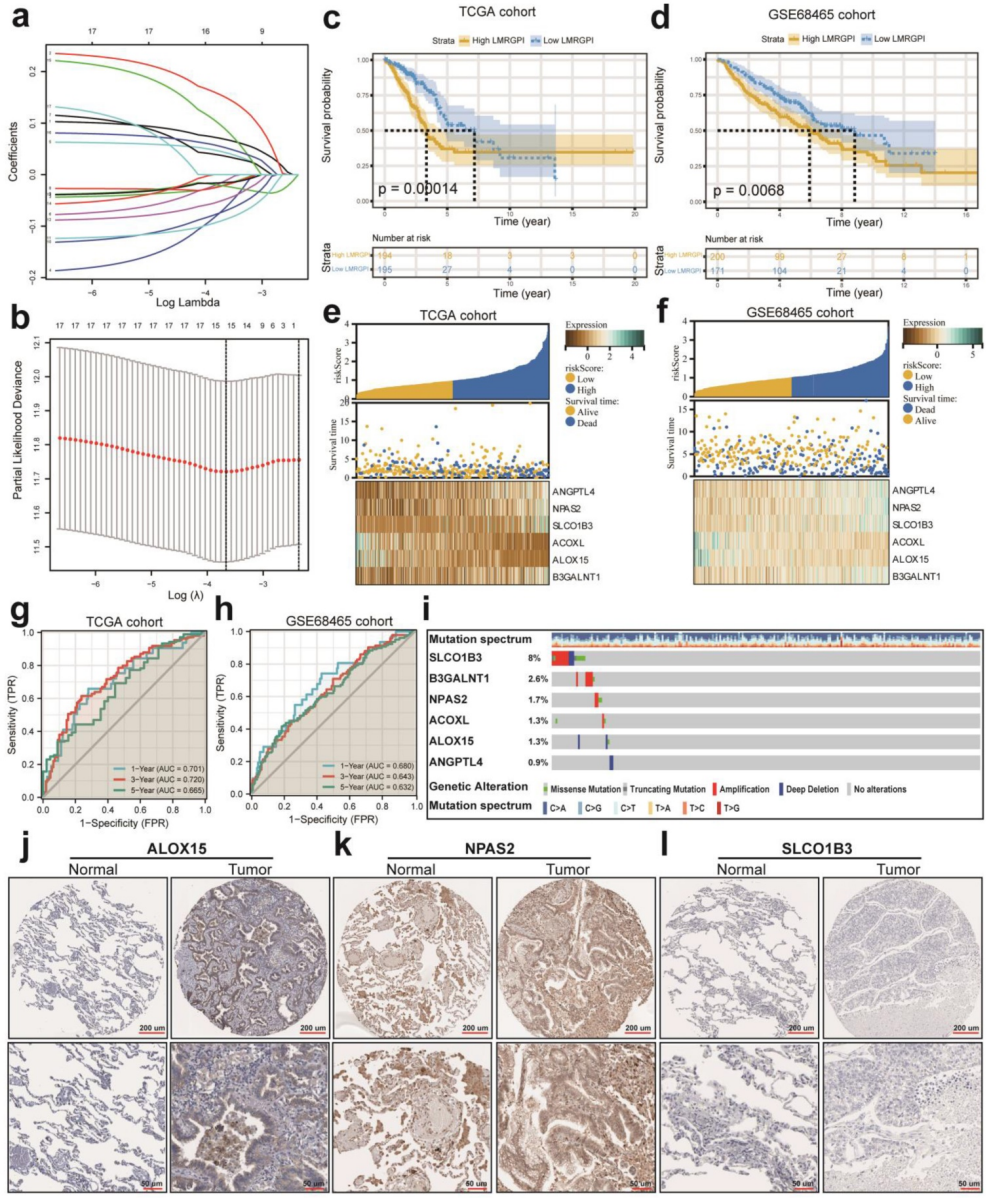
** LMRGPI establishment and validation for early-stage LUAD. (a)** Each independent variable's trajectory. The horizontal axis represents the log value of the independent variable lambda, and the vertical axis represents the independent variable's coefficient. **(b)** Confidence intervals with different values of lambda. **(c, d)** Survival curves to evaluate the risk stratification ability of LMRGPI in the TCGA-LUAD **(c)** and GSE68465 **(d)** cohorts. **(e, f)** Risk plots to illustrate the survival status of different LMRGPI groups in the TCGA-LUAD **(e)** and GSE68465 **(f)** cohorts. **(g, h)** ROC curves to evaluate the sensitivity and specificity of LMRGPI to predict the 1-, 3-, and 5- year OS of early-stage LUAD. in the TCGA-LUAD **(g)** and GSE68465 **(h)** cohorts. **(i)** mutation landscape of the identified prognostic LMRGs in the cBioPortal database. **(j-l)** Protein expression analysis of ALOX15 **(j)**, NPAS2 **(k)**, and SLCO1B3 **(l)** in LUAD and normal samples using the HPA database. LMRGPI, lipid metabolism-related gene prognostic index; LUAD, lung adenocarcinoma; LMRGs, lipid metabolism-related genes; LASSO, Least Absolute Shrinkage and Selection Operator; TCGA, The Cancer Genome Atlas; ROC, receiver operating characteristic curve; OS, overall survival; HPA, Human Protein Atlas.

First, we performed univariate Cox regression analysis to identify potential prognostic LMRGPI for early-stage LUAD in the TCGA-LUAD cohort. We found that 17 genres were correlated with the prognosis of these patients. Second, we conducted LASSO regression analysis to reduce the number of genes in the final risk model through R software, “glmnet” package, with 15 genes were identified through this step (**Figure 4a, b**). Ultimately, six genes were recognized as independent prognostic LMRGs via multivariate Cox analysis, including *ANGPTL4*, *NPAS2*, *SLCO1B3*, *ACOXL*, *ALOX15*, and *B3GALNT1*. According to their coefficients, we calculated LMRGPI using the following formula: LMRGPI= expression level of *ANGPTL4* * 0.108 + expression level of NPAS2* 0.265 + expression level of *SLCO1B3* * 0.083 + expression level of *ACOXL* * (-0.261) + expression level of *ALOX15* *(-0.191) + expression level of *B3GALNT1* * 0.177. All patients in this cohort were divided into high- and low-LMRGPI groups according to the median value of LMRGPI. The survival curve showed that patients with high-LMRGPI were associated with the worse OS when compared with patients with low-LMRGPI (**Figure 4c**). The risk plot also showed detailed survival outcomes of each patient (**Figure 4e**). We used the GSE68465 cohort as an independent external validation cohort to further assess the performance of LMRGPI. Consistently, similar results were observed in the GSE68465 cohort (**Figure 4d, f**). Besides, we used ROC curves and calculated AUC values to evaluate the performance of LMRGPI in predicting 1-, 3-, and 5-year OS of early-stage LUAD patients. We observed that LMRGPI had good performance in predicting the OS in these individuals both in the TCGA-LUAD cohort (AUC for 1-, 3-, and 5-year OS: 0.701, 0.720, and 0.665; **Figure 4g**) and GSE68465 cohort (AUC for 1-, 3-, and 5-year OS: 0.680, 0.643, and 0.632; **Figure 4h**).

**Figure 5:**
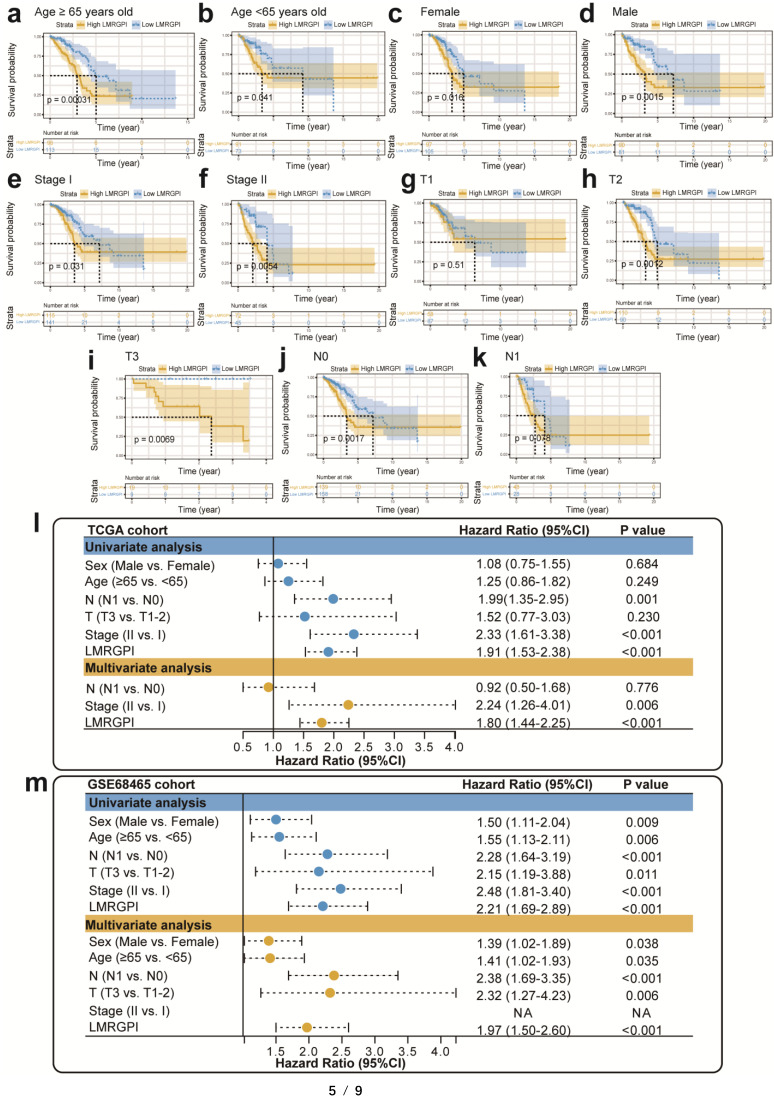
** Subgroup analysis and independent prognostic analysis of LMRGPI. (a-k)** Subgroup analysis stratified by age **(a, b)**, gender **(c, d)**, disease stage **(e, f)**, T stage **(g-i)**, and N stage **(j, k)** to further confirm the risk stratification ability of LMRGPI in different subgroups. **(l, m)** The univariate **(l)** and multivariate **(m)** Cox analyses to determine the independent prognostic ability of LMRGPI. LMRGPI, lipid metabolism-related gene prognostic index.

Next, we used the cBioPortal database to summarize the mutation landscape of the identified LMRGs in the multivariate Cox analysis. We observed that 8% of patients harbored *SLCO1B3* mutation, with amplification being the most predominant genetic alteration type (**Figure 4i**). Meanwhile, the HPA database was also adopted to analyze their protein expression level. We found that ALOX15 and NPAS2 protein were highly expressed in the LUAD samples (**Figure 4j, k**). In contrast, SLCO1B3 protein was not detected in the LUAD sample (**Figure 4l**). The protein expression level of other prognostic LMRGs is not available in the HPA database. Subsequently, we performed subgroup analysis to evaluate the prognostic significance of LMRGPI in different subgroups, including age (**Figure 5a, b**), gender (**Figure 5c, d**), disease stage (**Figure 5e, f**), T stage (**Figure 5g-i**), and N stage (**Figure 5j, k**). It indicated that except for patients with T1 (**Figure 5g**) and N1 (**Figure 5k**) stage disease, higher LMRGPI was significantly associated with poor OS in other subgroups. Ultimately, we performed single factor and multi-factor Cox analyses to determine whether LMRGPI could be an independent prognostic factor for early-stage LUAD compared with other common clinicopathological parameters. Not surprisingly, we observed that LMRGPI could serve as an independent prognostic index for these individuals (**Figure 5l, m**).

### Nomograms development and assessment

**Figure 6:**
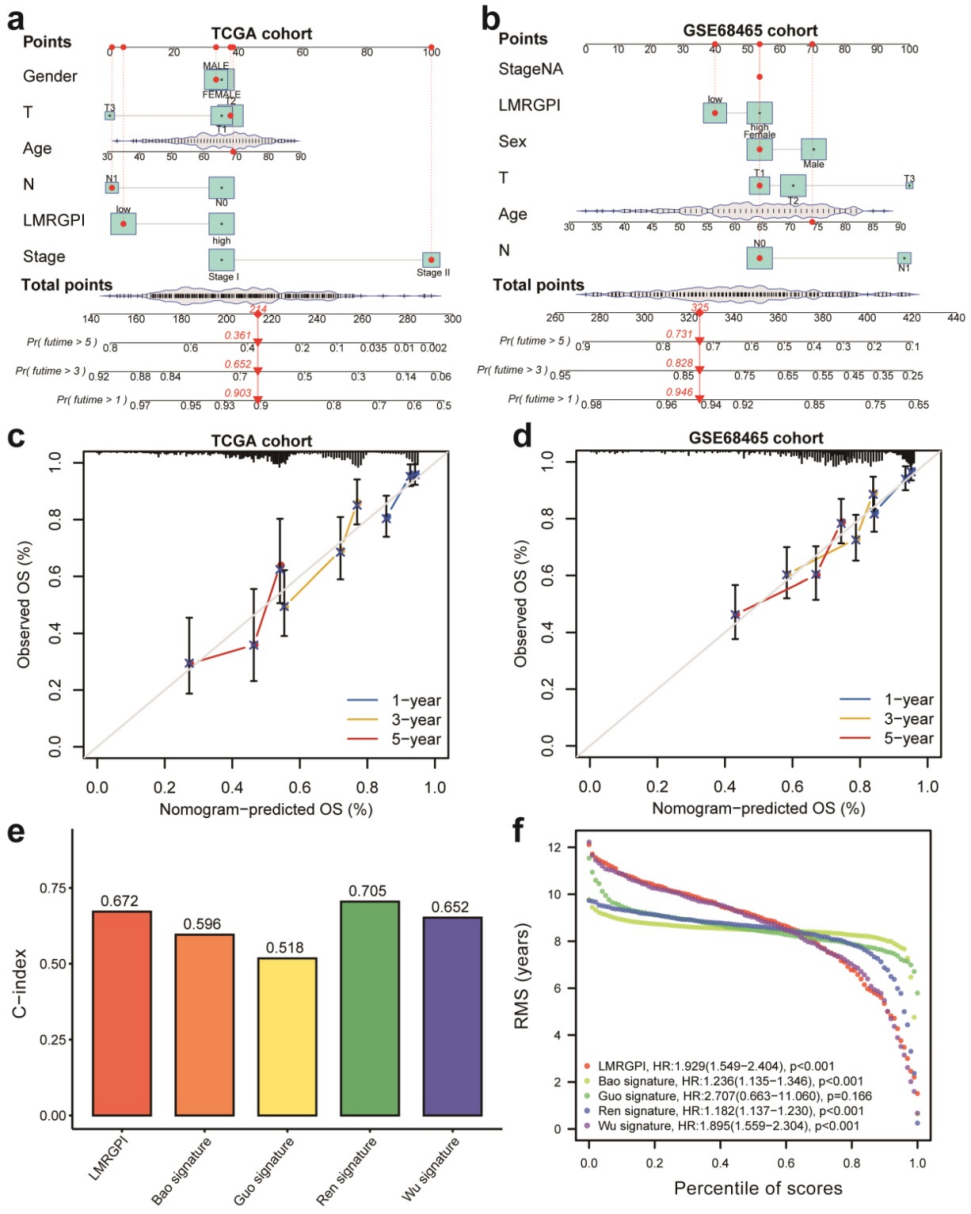
** Nomogram construction and validation to predict the prognosis of early-stage LUAD. (a, b)** Two nomograms were constructed via R software, “rms” and “regplot” packages to present the survival probability of each patient in the TCGA-LUAD **(a)** and GSE68465 **(b)** cohorts. **(c)** Calibration curve to evaluate the consistency between the actual and predicted 1-, 3- and 5-year OS of early-stage LUAD in the TCGA cohort. **(d)** Calibration curve to evaluate the consistency between the actual and predicted 1-, 3- and 5-year OS of early-stage LUAD in the GSE68465 cohort. **(e, f)** The C-index **(e)** and RMS **(f)** values of LMRGPI and other prognostic models were calculated to compare their predictive ability. LUAD, lung adenocarcinoma; TCGA, The Cancer Genome Atlas; OS, overall survival.

Next, we developed two nomograms to illustrate each patient's 1-, 3-, and 5-year survival probability by integrating LMRGPI and common clinicopathological variables. We could easily calculate each patient's total points and the corresponding survival probability using the constructed nomogram (**Figure 6a, b**). Calibration curves indicated higher consistencies between the predicted OS and the actual OS rates in the TCGA-LUAD cohort (**Figure 6c**) and the GSE68465 cohort (**Figure 6d**). Furthermore, we compared the discrimination ability of LMRGPI and other preexisting prognostic scores in predicting the OS of early-stage LUAD. It showed that LMRPI had comparable risk stratification ability to other prognostic scores (**Figure S1b-f**). The C-index (**Figure 6e**) and RMS (**Figure 6f**) values also supported the above results.

### Clinical relevance, mutation landscape, and enrichment analysis between high- and low- LMRGPI groups

**Figure 7:**
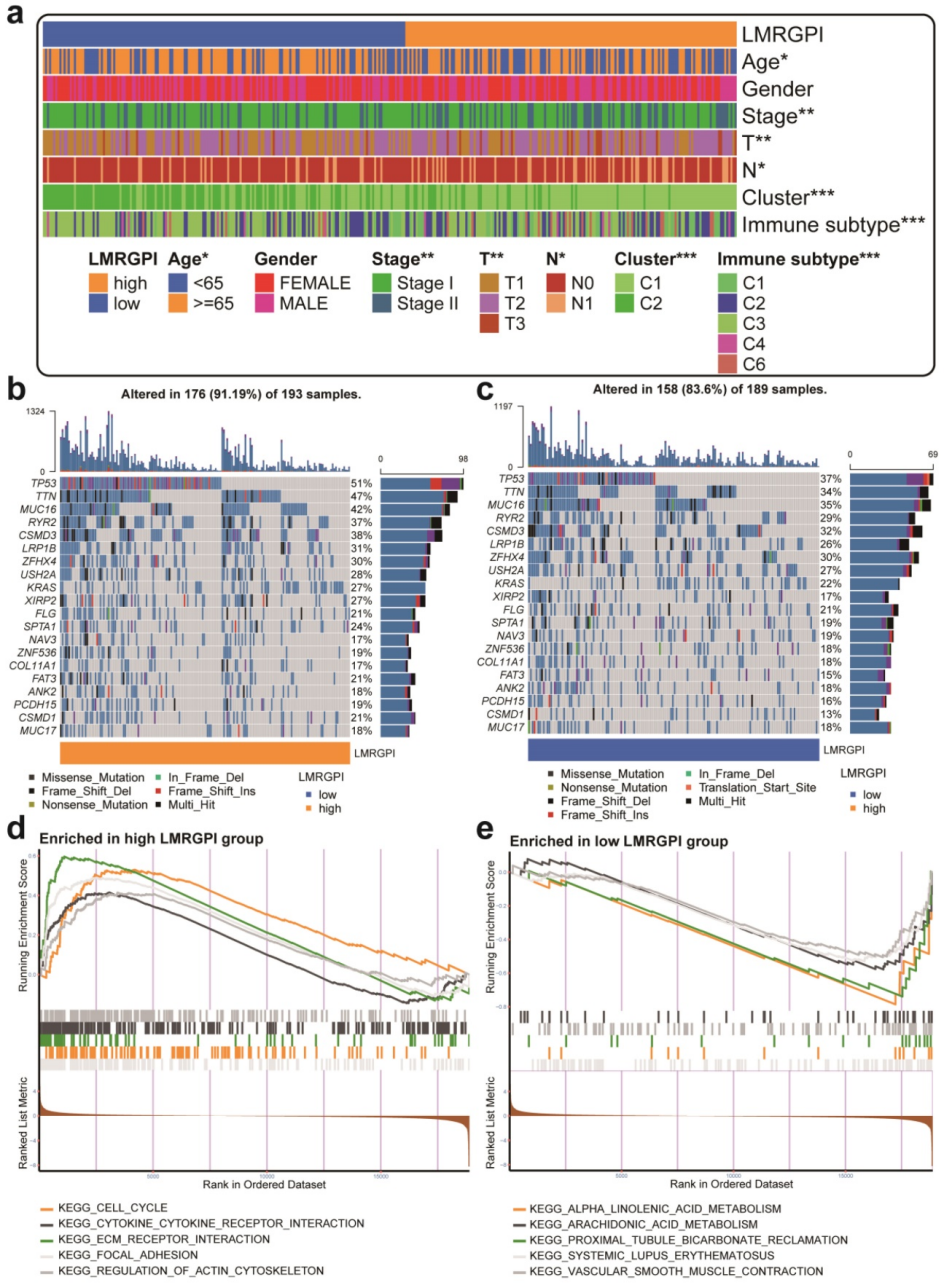
** Clinical relevance, gene mutation landscape, and pathway enrichment analysis between different LMRGPI groups. (a)** The heatmap was generated to show the relationship between LMRGPI and other clinicopathological variables. **(b, c)** Waterfall plots summarize the gene mutation landscape in high- **(b)** and low-LMRGPI **(c)** groups. **(d, e)** GSEA to investigate the biological processes and pathways enriched in high- (d) and low-LMRGPI **(e)** groups. LMRGPI, lipid metabolism-related gene prognostic index; GSEA, Gene Set Enrichment Analysis; * represents *P*<0.05; ** represents *P*<0.01; *** represents *P*<0.001.

Next, we investigated the relationship between LMRGPI and clinicopathological characteristics, different clusters, and immune subtypes. It showed that LMRGPI was significantly correlated with age, disease stage, T stage, N stage, cluster, and immune subtype (**Figure 7a**). Afterward, we generated two waterfall plots to explore the detailed gene mutation characteristics between high- and low-LMRGPI groups. We identified that *TP53*, *TTN*, *MUC16* were the most frequently mutated genes in these groups (**Figure 7b, c**). Besides, we also observed that the high-LMRGPI group harbored a more frequent *TP53* mutation rate than the low-LMRGPI group (**Figure 7b, c**). Furthermore, we performed GSEA analysis to identify the most significantly enriched pathways between the two groups. We found that genes in the high-LMRGPI significantly enriched in cell cycle, cytokine-cytokine receptor interaction, ECM receptor interaction, focal adhesion, and regulation of actin cytoskeleton (**Figure 7d, Table S1**). However, genes in the low-LMRGPI significantly enriched in alpha-linolenic acid metabolism, arachidonic acid metabolism, proximal tubule bicarbonate reclamation, systemic lupus erythematosus, and vascular smooth muscle contraction (**Figure 7e, Table S1**).

### The immune function between high- and low-LMRGPI groups

**Figure 8:**
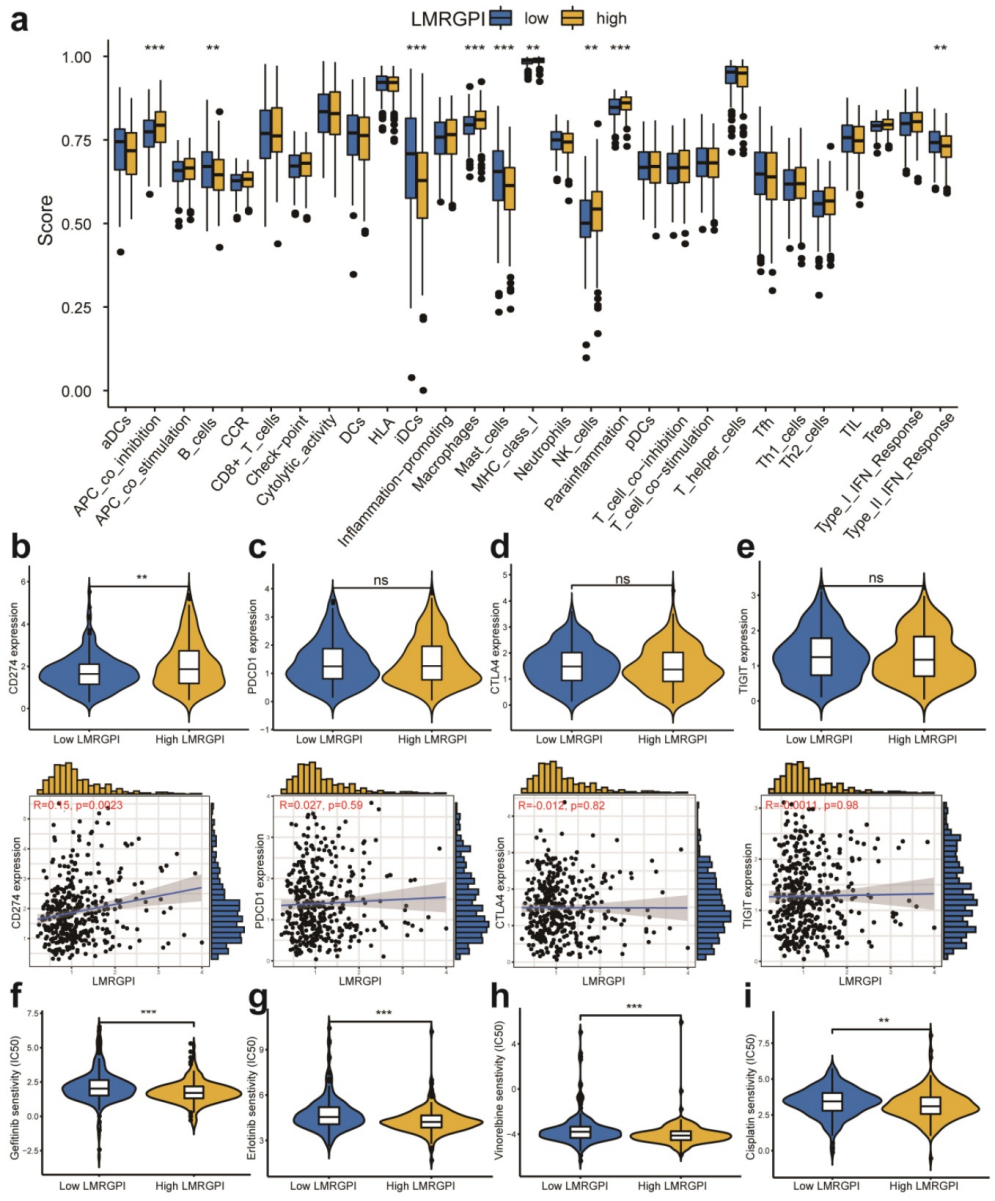
** Immune function, ICIs related genes expression pattern, and chemotherapeutic drugs sensitivity between different LMRGPI groups. (a)** Immune cells infiltration score and immune-related pathways activity in the low- and high-risk groups estimated by ssGSEA. **(b-e)** The correlation between LMRGPI and the expression level of *CD274* **(b)**, *PDCD1* **(c)**, *CTLA4* **(d)**, and *TIGIT* **(e)**. **(f-i)** The relationship between LMRGPI and drug sensitivity of gefitinib** (f)**, erlotinib** (g)**, vinorelbine **(h)**, and cisplatin** (i)**. ICIs, immune checkpoint inhibitors; LMRGPI, lipid metabolism-related gene prognostic index; ssGSEA, single-sample gene set enrichment analysis; ns represents no statistical significance; ** represents *P*<0.01; *** represents *P*<0.001.

We then adopted ssGSEA to estimate the infiltrating score of immune cells and the activity of immune-related pathways in different LMRGPI groups. The results demonstrated that the infiltration levels of B cells, iDCs, Macrophages, Mast cells, and NK cells were significantly different in the two groups (**Figure 8a**). Meanwhile, the two groups also had different scores of APC co-inhibition, MHC class I, parainflammation, and Type II IFN response (**Figure 8a**). Subsequently, we investigated the correlation between LMRGPI and immune cells infiltration, TMB value, and the expression level of common ICIs related genes. The results revealed that LMRGPI was positively correlated with the infiltration levels of cytotoxic lymphocytes and fibroblasts. In contrast, it was negatively correlated with the infiltration levels of T cells, myeloid dendritic cells, neutrophils, and endothelial cells (**Figure S2a**). Besides, we observed that LMRGPI was positively correlated with TMB value (**Figure S2b**). We found that higher LMRGPI was also significantly associated with up-regulation of *CD274* (**Figure 8b and Figure S2c**). Nevertheless, there was no significant statistical difference between LMRGPI and *PDCD1* (**Figure 8c**), *CTLA4* (**Figure 8d**), *TIGIT* (**Figure 8e**), and *LAG3* (**Figure S2d**) expression. Interestingly, it showed that LMRGPI also positively correlated with *POLE2* expression (**Figure S2e**).

### Chemotherapeutic and immunotherapy response rates between high- and low-LMRGPI groups

**Figure 9:**
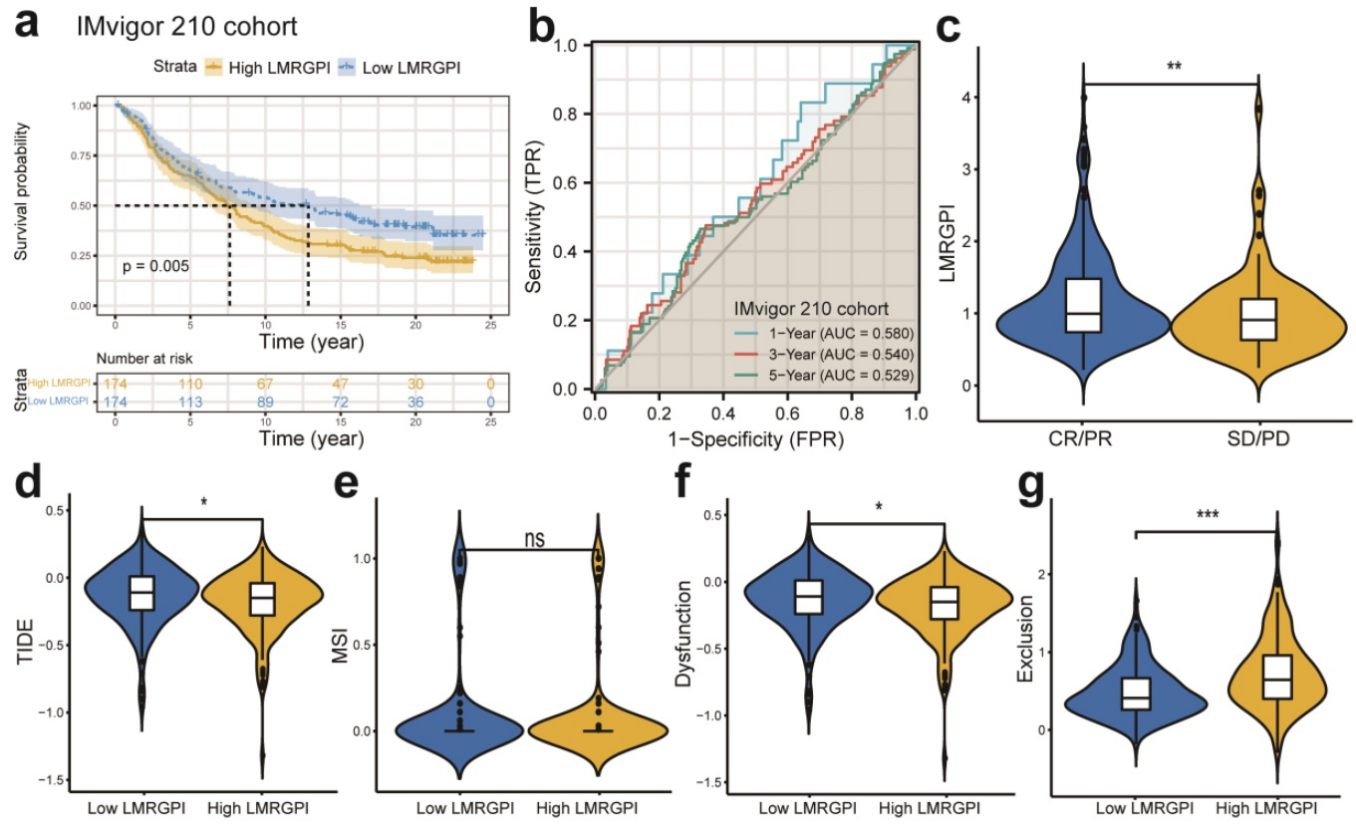
** The prognostic value of LMRGPI in ICIs treatment. (a)** Kaplan-Meier survival analysis of the LMRGPI subgroups in IMvigor 210 cohort. **(b)** ROC curves to show the prognostic value of LMRGPI in predicting the 1-, 3-, and 5-year OS of patients treated with ICIs in the IMvigor 210 cohort. **(c)** The relationship between LMRGPI and immunotherapy response in the TCGA-LUAD cohort. **(d-g)** The correlation between LMRGPI and TIDE score **(d)**, MSI score **(e)**, immune dysfunction score **(f)**, and immune exclusion score **(g)**. LMRGPI, lipid metabolism-related gene prognostic index; ICIs, immune checkpoint inhibitors; ROC, receiver operating characteristic curve; OS, overall survival; TCGA, The Cancer Genome Atlas; LUAD, lung adenocarcinoma; CR, complete response; PR, partial response; SD, stable disease; PD, progressive disease; ns represents no statistical significance; * represents *P*<0.05; ** represents *P*<0.01; *** represents *P*<0.001.

Next, we investigated the association between LMRGPI and commonly used antitumor drugs sensitivity via R software, “pRRophetic” package to evaluate the clinical utility of LMRGPI for the treatment of early-stage LUAD. We found that lower LMRGPI was significantly correlated with higher IC50 of gefitinib (**Figure 8f**), erlotinib (**Figure 8g**), cisplatin (**Figure 8h**), and vinorelbine (**Figure 8i**). We also downloaded the gene expression matrix and survival data of the IMvigor 210 cohort to explore the prognostic value of LMRGPI in predicting the OS of patients treated with ICIs. It revealed that LMRGPI could also be served as a potential prognostic biomarker for these patients (**Figure 9a, b**). Besides, we used the TIDE algorithm to predict the response rate of immunotherapy in high- and low-LMRGPI groups. We observed that patients with higher immunotherapy responses presented with higher LMRGPI (**Figure 9c**). Ultimately, we explored the correlation between LMRGPI and TIDE score, MSI, immune exclusion score, and immune dysfunction score. The results demonstrated that lower LMRGPI was significantly associated with a high TIDE score (**Figure 9d**) and immune dysfunction score (**Figure 9f**). However, higher LMRGPI was correlated with a higher immune exclusion score (**Figure 9g**). There was no significant difference between LMRGPI and MSI (**Figure 9e**).

## Discussion

The current study identified two different LMRGs subtypes based on the NMF algorithm and explored their association with patients' prognosis and immune cells infiltration. We observed very different prognostic and immune profiles between different subtypes. Most importantly, we developed a novel prognostic index, LMRGPI, based on the expression levels of six LMRGs. It could be used to predict the prognosis and treatment response of early-stage LUAD patients. Furthermore, the results from an independent external validation cohort validation also depict a similar predictive ability of LMRGPI.

We observed that patients in cluster 1 suffered from worse OS, PFS, and DFS than patients in cluster 2. Besides, it showed that patients in cluster 1 are mainly classified into Immune C1, Immune C2, and Immune C6 subtypes, which are correlated with more aggressive immune infiltrates and worse prognosis 17, 22. On the contrary, patients in cluster 2 are mainly classified into the Immune C3 subtype, which is associated with a more favorable immune composition and better clinical outcomes 17, 22. Therefore, we furtherly estimated the infiltration of the immune cells in different clusters. We found that cluster 1 correlated with higher cytotoxic lymphocytes, fibroblasts, and NK cells infiltration levels than cluster 2. Accumulating studies have shown that cancer-associated fibroblasts (CAFs) could transfer lipid to the TME to support cancer cell growth 15, 23, 24. Recently, Gong et al. elucidated that reprogramming of lipid metabolism in CAFs potentiates migration of colorectal cancer cells through *in vivo* and *in vitro* experiments 15. Furthermore, dysfunctional CD8+ T cells increased their uptake and accumulation of specific long-chain fatty acids (FAs), thus resulting in T cell dysfunction, inhibition of mitochondrial function, and reduction of FA catabolism 14, 25. Lipids also affect cytotoxic NK cells, which are vital in the antitumor response 14, 26. A recent study revealed that lipid accumulation in NK cells attenuated its antitumor immunity and failed to reduce tumor growth in obesity 27. Therefore, lipid accumulation could reprogram immune cells in TME and support a tumor-promoting microenvironment.

Next, six LMRGs are recognized as correlated with OS of early-stage LUAD through LASSO and Cox regression analyses, including *ANGPTL4*, *NPAS2*, *SLCO1B3*, *ACOXL*, *ALOX15*, and *B3GALNT1*. *ANGPTL4* is a member of the angiopoietin family and acts as a regulator of lipid and glucose metabolism. Upregulation of *ANGPTL4* is associated with malignant biological behavior in various malignancies 28-31. Yang et al. reported that *ANGPTL4* regulates ferroptosis through *NOX2*, thus inducing cell death and chemoresistance in epithelial ovarian cancer 28. *NPAS2* is the most significant circadian rhythm gene, has received extensive attention due to its sophisticated function in various diseases development. He et al. revealed that *NPAS2* polymorphism is an independent prognostic marker for lung cancer patients 32. Besides, Yuan et al. indicated that overexpression of *NPAS2* significantly promoted cell proliferation and inhibited mitochondria-dependent intrinsic apoptosis, and thus contributed to a worse prognosis of patients with liver cancer 33. *SLCO1B3* is a liver-specific transporter and is physiologically involved in the uptake of bile acids 34. Although numerous studies explored its functional change and prognostic value in various malignancies, the molecular regulatory mechanism of *SLCO1B3* is not well elucidated 35. Sekine et al. reported that the expression of *SLCO1B3* is associated with intratumoral cholestasis and *CTNNB1* mutations in liver cancer 34. *ACOXL* is a rate-limiting enzyme in peroxisomal fatty acids β-oxidation, and it could initiate the oxidative metabolism of long-chain fatty acids 36. Therefore, *ACOXL* plays a crucial role in lipid metabolism. He et al. found that *ACOXL* is overexpressed in prostate cancer cell lines and could be served as a novel biomarker for prostate cancer 37. Nevertheless, the biological function and prognostic significance of *ACOXL* in LUAD and other tumors are not studied 37. *ALOX15* oxidizes polyunsaturated fatty acids to generate several bioactive lipid metabolites, and many studies have elucidated its importance in oxidative and inflammatory responses 38. Recently, Zhang et al. revealed that CAFs could secrete exosomal miR-522 to inhibit ferroptosis in gastric cancer cell lines and promote acquired chemoresistance by targeting *ALOX15* and blocking lipid peroxides accumulation 39. *B3GALNT1* is a galactosyltransferase that catalyzes the transfer of galactose 40. Umeyama et al. indicated that *B3GALNT1* is a potential therapeutic target in lung cancer through bioinformatic analysis 40. However, the association between *B3GALNT1* expression and cancer development and progression is not well discussed as well.

Subsequently, all patients were divided into low- and high-LMRGPI groups based on the median value of risk score both in TCGA and GSE68465 cohorts. Subsequent analyses demonstrated that it could be used as an independent prognostic index for early-stage LUAD. Ultimately, we constructed two nomograms to predict each patient's 1-, 3-, and 5-year survival probability by integrating LMRGPI with other clinicopathological variables, with a series of tests being performed to evaluate their discrimination and calibration abilities. These results proved that LMRGPI is a reliable prognostic index, and the nomograms could be an effective tool to predict the prognosis of early-stage LUAD. We then investigated the gene mutation landscape, immune function, and treatment response in different LMRGPI groups. We identified that the high-LMRGPI group harbored a more frequent TP53 mutation rate than the low-LMRGPI group. Numerous studies identified that *TP53* mutation is closely correlated with treatment resistance and lethal prognosis in lung cancer 41-43. However, many studies revealed that *TP53* mutation was significantly correlated with remarkable clinical benefit from *PD-1* inhibitors for patients with LUAD since it increases TMB, up-regulates *PD-L1* expression, and remodels TME 43-45.

In this study, we also evaluated the relationship between LMRGPI and chemotherapeutics efficacy, suggesting that lower LMRGPI was correlated with the sensitivity to vinorelbine and cisplatin and the first-generation epidermal growth factor receptor (EGFR) tyrosine kinase inhibitors (TKIs) (gefitinib and erlotinib). The results of lung adjuvant cisplatin evaluation (LACE) meta-analysis confirmed that adjuvant cisplatin plus vinorelbine can significantly improve the OS of early-stage LUAD after the operation 46. Because EGFR was frequently mutated in patients with LUAD, especially in Eastern Asia, numerous studies have attempted to apply EGFR-TKIs in early-stage LUAD treatment. The results from the ADAURA study reported that adjuvant osimertinib (a third-generation EGFR-TKI) could significantly prolong the DFS of resectable NSCLC with EGFR mutation 47. Besides, the EVIDENCE study also revealed that icotinib could significantly improve DFS and has a better tolerability profile in these patients 48. Our study provided a novel prognostic index that could stratify patients who may benefit from adjuvant chemotherapy or targeted therapy. With the promising effect of ICIs in advanced/ metastatic lung cancer treatment, more and more studies are investigating the possibility of immunotherapy in early-stage lung cancer. IMpower010 is a randomized multicentre phase 3 study that explores adjuvant atezolizumab (a* PD-L1* inhibitor) versus best supportive care in early-stage NSCLC 49. The results showed that atezolizumab after adjuvant chemotherapy offers a promising treatment option for these patients 49. Given that *PD-L1* and TMB are the most predominantly used biomarkers to predict the efficacy of immunotherapy in lung cancer, higher values predict better therapeutic efficacy. We investigated the relationship between LMRGPI and TMB value and *PD-L1* expression. The results revealed that LMRGPI was positively correlated with TMB value and *CD274* expression level. Besides, we used the TIDE algorithm to predict the response rate of immunotherapy in high- and low-LMRGPI groups. A lower TIDE score means a lower potential for immune evasion, suggesting patients may benefit from ICIs treatment 50. We observed that lower LMRGPI was significantly associated with a higher TIDE score and immune dysfunction score, indicating an immune dysfunction status. Our study showed that higher LMRGPI was associated with superior immunotherapy efficacy in early-stage LUAD, and it could be a novel biomarker to ICIs efficacy prediction. However, there are several inevitable limitations in our study. First, although LMRGPI could effectively predict the OS and treatment response of early-stage LUAD and an independent external cohort was used to validate its performance, all these results were obtained from the bioinformatic analysis. Second, we identified that CAFs were significantly correlated with LMRGPI and its infiltration level differed in two subtypes. However, these results are observed based on algorithm estimation. Third, searching for effective prognostic and predictive biomarkers for immunotherapy is an arduous task for us and needs a long way to go. Our study developed a novel biomarker and provided potential insights in this area. However, well-designed prospective studies are warranted in the future to address this issue.

## Conclusions

To sum up, lipid metabolism plays a crucial role in the prognosis, TME, and antitumor immune response of early-stage LUAD. We identified two distinct population subtypes according to LMRGs, and they have very different prognoses and immune functions. Most importantly, we established a novel biomarker LMRGPI that could predict the OS and treatment response of these individuals. Taken together, LMRGPI is a promising biomarker for early-stage LUAD patients.

## Supplementary Material

Supplementary figures.

Supplementary table.

## Figures and Tables

**Table 1:** The detailed clinical characteristics of patients in the TCGA and GEO cohorts

Variables	TCGA cohort	GSE68465 cohort
**Age**		
≥65	218	191
<65	170	180
Unknown	15	0
**Gender**		
Female	219	188
Male	184	183
**Clinical stage**		
I	279	114
II	124	257
**T stage**		
T1	154	138
T2	220	217
T3	29	16
**N stage**		
N0	314	292
N1	82	79
Unknown	7	0
**Survival status**		
Death	123	175
Alive	280	196

## Abbreviations

LUAD: lung adenocarcinoma
NCCN: National Comprehensive Cancer Network
TME: tumor microenvironment
LMRGs: lipid metabolism-related genes
LMRGPI: lipid metabolism-related gene prognostic index
NMF: non-negative Matrix Factorization
PFS: progression-free survival
DFS: disease-free survival
GEO: Gene Expression Omnibus
DELMRGs: differentially expressed lipid metabolism-related genes
KEGG: Kyoto Encyclopedia of Genes and Genomes
GO: Gene Ontology
BP: biological process
CC: cellular component
MF: molecular function
LASSO: Least Absolute Shrinkage and Selection Operator
ROC: receiver operating characteristic
HPA: Human Protein Atlas
IHC: immunohistochemistry
GSEA: Gene set enrichment analysis
ssGSEA: single-sample gene set enrichment analysis
TMB: tumor mutation burden
ICIs: immune checkpoint inhibitors
AUC: area under the curve
TIDE: Tumor Immune Dysfunction and Exclusion
NSCLC: non-small cell lung cancer
MSI: microsatellite instability
CAFs: cancer-associated fibroblasts
FAs: fatty acids
EGFR: epidermal growth factor receptor
TKIs: tyrosine kinase inhibitors
LACE: lung adjuvant cisplatin evaluation
