# Supplementary material for: Lipid metabolism-related gene prognostic index (LMRGPI) reveals distinct prognosis and treatment patterns for patients with early-stage pulmonary adenocarcinoma
Source: Int J Med Sci. 2022 Mar 28;19(4):711–28. doi: 10.7150/ijms.71267 (PMC9108406; doi:10.7150/ijms.71267)
Supplement: Supplementary file 1 — Supplementary figures. [file ijmsv19p0711s1.pdf]

Figure S1

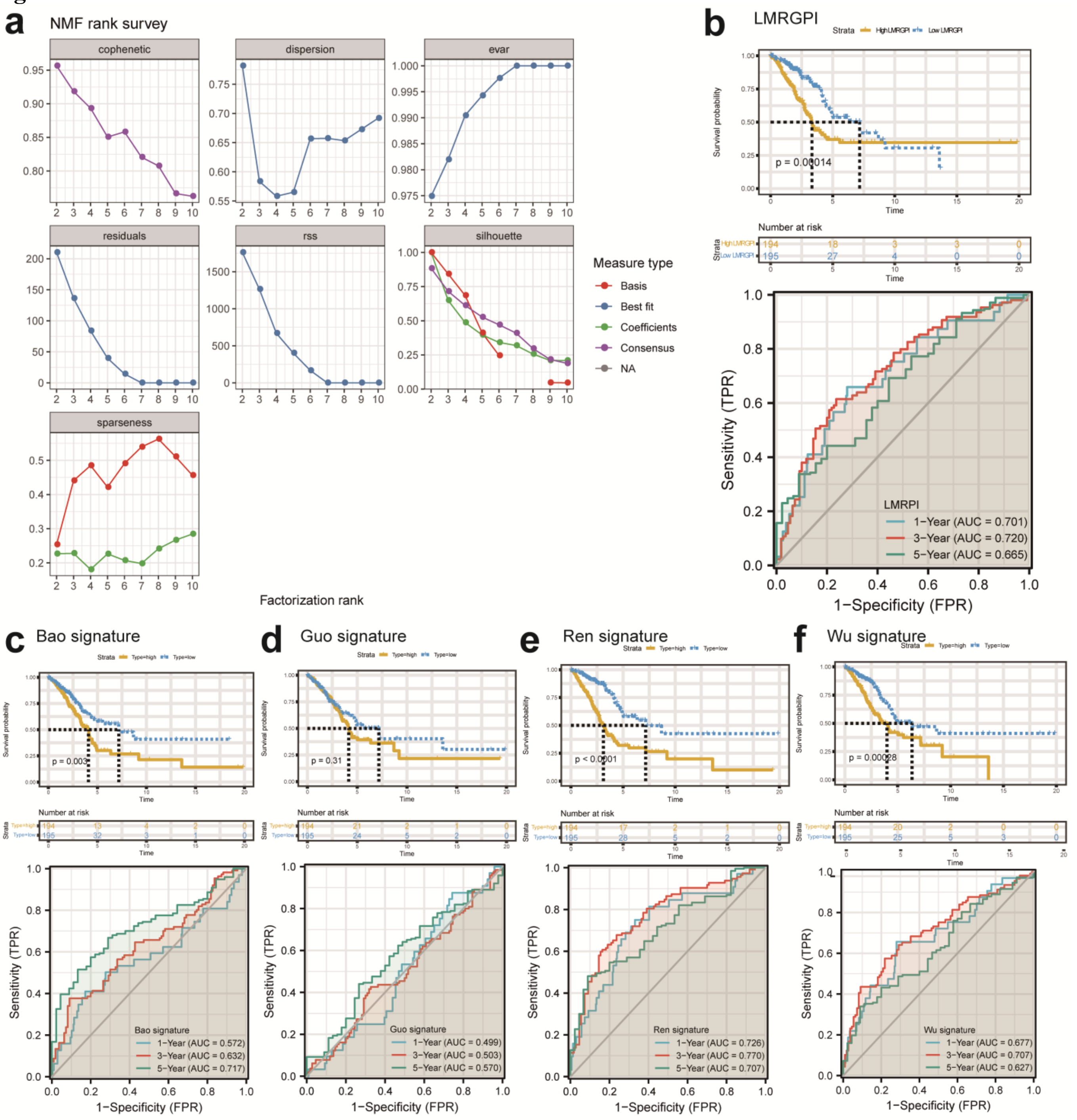

Figure S1. **(a)** The process of subtypes classification using NMF. **(b-f)** Kaplan-Meier survival analysis and ROC analysis of LMRGPI and other pre-existing prognostic models in predicting the OS of early-stage LUAD. NMF, non-negative Matrix Factorization; ROC, receiver operating characteristic curve; LMRGPI, lipid metabolism-related gene prognostic index; LUAD, lung adenocarcinoma.

Figure S2

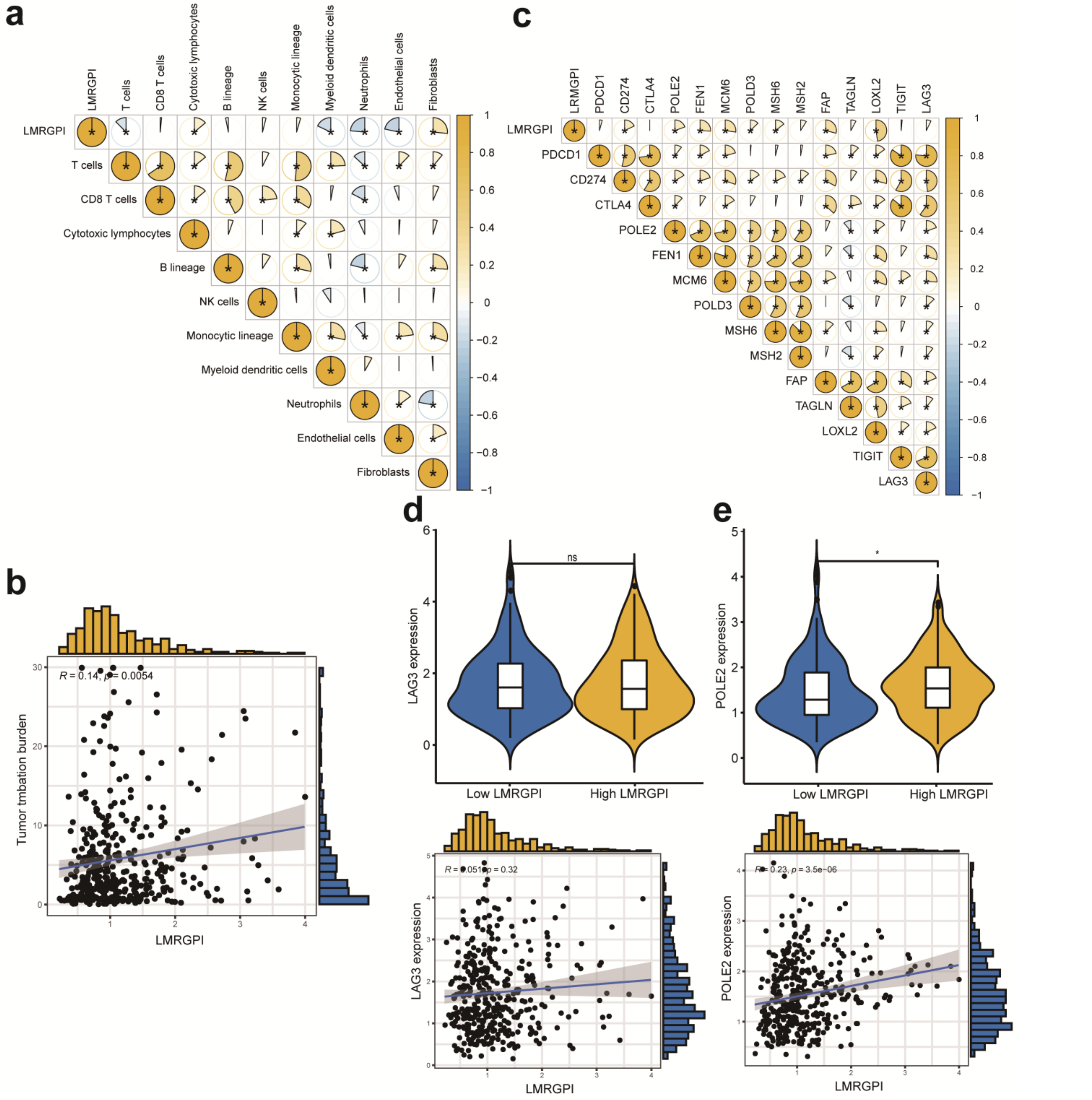

Figure S2. **(a)** The correlation between LMRGPI and the infiltration of the immune cells. **(b)** The association between LMRGPI and ICIs related genes expression. **(c)** The relationship between LMRGPI and TMB. **(d-e)** Box plots and correlation plots to show the relationship between LMRGPI and *LAG3* **(d)** and *POLE2* **(e)**. ns represents no statistical significance; * represents *P*<0.05.
